# Using Multiple Diagnostic Methods for Occupational Asthma Assessment

**DOI:** 10.1111/crj.70197

**Published:** 2026-05-13

**Authors:** Bilge Akgündüz, Günhan Yavaşoğlu

**Affiliations:** ^1^ Occupational Diseases Clinic Eskişehir City Hospital Eskişehir Turkey; ^2^ Chest Diseases Clinic Eskişehir City Hospital Eskişehir Turkey

**Keywords:** methacholine PC20 variability, misdiagnosis, occupational asthma, peak expiratory flow rate monitoring

## Abstract

**Introduction:**

Occupational asthma (OA) is a significant work‐related respiratory disorder requiring accurate diagnostic methods. This study evaluates the sensitivity and specificity of PC20 variability in OA diagnosis and compares it with peak expiratory flow rate (PEFR) monitoring.

**Method:**

A retrospective descriptive study was conducted. Fifty‐one individuals with suspected OA were assessed. Pulmonary function tests, PEFR monitoring, and methacholine challenge test were performed. PC20 variability was analyzed by comparing values during workplace exposure and after exposure cessation.

**Results:**

A total of 51 suspected occupational asthma (OA) cases were evaluated (mean age: 38.14 ± 8.10 years, 68.6% male). The most common symptom was shortness of breath (mean duration: 19.61 ± 29.23 months), with an average occupational exposure of 7.34 ± 6.82 years, primarily to low molecular weight (LMW) agents. OA was diagnosed in 70.6% (*n* = 36) of cases, with longer symptom duration and exposure time than nonoccupational asthma. OA cases had longer symptom duration (22.50 ± 32.98 months vs. 12.67 ± 16.08 months in non‐OA cases) and slightly longer occupational exposure (7.51 ± 5.94 years vs. 6.92 ± 8.83 years).

PC20 (provocative concentration of methacholine causing a 20% fall in FEV_1_) variability was analyzed. IgE‐mediated asthma was found in 16.7% of LMW‐exposed cases, whereas nonimmunologic mechanisms accounted for 83.3%. PC20 levels were lower during exposure but improved after exposure cessation. PC20 variability was positive in 77.8% (n:28) of cases and had a sensitivity of 90% and a specificity of 92%. In 13.9% of OA cases, methacholine challenge test could not be performed due to low FEV1 levels, and diagnoses were made based on PEFR variability. If PC20 variability was not performed, PEFR variability alone could lead to misclassification in 22.2% (n:8) of cases. The majority of OA cases were associated with the metal, foundry, and ceramics industries, with inorganic dust exposure as the most common exposure (27.8%).

**Conclusion:**

PC20 variability is a highly sensitive and specific tool for diagnosing OA and may help reduce misdiagnosis associated with PEFR‐based assessments. However, in patients where the methacholine challenge test is not feasible, PEFR variability remains a valuable alternative for diagnostic evaluation. Integrating PC20 variability into diagnostic protocols can enhance accuracy and improve patient management.

## Graphical abstract

PC20 variability demonstrated higher diagnostic accuracy than PEFR monitoring in occupational asthma (OA), with a sensitivity of 90% and specificity of 92%. PEFR alone resulted in a 22.2% misclassification rate. Incorporating PC20 variability into diagnostic evaluation may reduce misdiagnosis and improve clinical outcomes in high‐exposure settings.

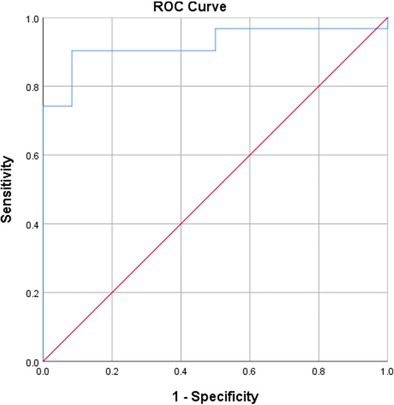

## 1 Introduction

The specific bronchial provocation test (SBPT) is considered the gold standard for diagnosing occupational asthma and can be performed in hospital laboratories or workplace settings. However, due to the need for controlled environments, specialized equipment, and experienced personnel, SBPT is only available in a limited number of facilities worldwide, making it available only in a limited number of centers worldwide, thereby limiting accessibility [1, 2]. Workplace‐based SBPT is often impractical due to logistical and occupational constraints [3]. Consequently, alternative diagnostic methods are frequently employed, particularly in resource‐limited settings.

One commonly used alternative is peak expiratory flow rate (PEFR) monitoring, which requires measuring PEFR at least four times daily over 2 weeks, both during and outside work. A variability greater than 10% between work and nonwork periods suggests occupational asthma [4, 5]. However, PEFR results can be influenced by patient effort and reporting bias, particularly in cases where job security is a concern [6]. In Turkey, legislation mandates PEFR monitoring for occupational asthma diagnosis, emphasizing its clinical relevance [7].

Another diagnostic tool, the methacholine nonspecific bronchial provocation test (NSBPT), also known as the methacholine challenge test, is used in some tertiary hospitals under controlled conditions. It has higher sensitivity in detecting bronchial hyperresponsiveness [8, 9, 10] and provides more objective results than PEFR due to automated reporting and supervision by trained personnel [11].

In this context, methacholine challenge testing provides a direct and controlled bronchoconstrictive stimulus, allowing standardized assessment of airway hyperresponsiveness under supervised conditions. In contrast, PEFR monitoring reflects variations in airway function during natural workplace exposure, which is inherently variable and lacks standardization. Therefore, methacholine challenge testing offers a more objective and reproducible assessment of bronchial responsiveness.

Additional diagnostic tools, including specific IgE testing, skin prick testing, fractional exhaled nitric oxide (FeNO), and sputum eosinophil analysis, may support the diagnosis; however, their sensitivity and specificity are variable and often limited, particularly in occupational asthma [12, 13, 14]. Therefore, these tests should be considered supportive rather than definitive diagnostic tools.

Importantly, a negative result in any single test, including SBPT, does not exclude occupational asthma, given the multifactorial nature of its pathogenesis [15].

According to current European Respiratory Society (ERS) guidelines, asthma diagnosis may be established based on a positive result from a single objective test, such as bronchodilator reversibility, PEFR variability, or FeNO. Nevertheless, in occupational asthma, a combined diagnostic approach is often recommended to improve diagnostic accuracy [1, 16, 17].

This study aims to compare the diagnostic performance of PEFR monitoring and the methacholine challenge test in patients with suspected occupational asthma referred to an occupational disease clinic.

## 2 Methods

This study was designed as a retrospective descriptive analysis to investigate the occurrence and diagnosis of occupational asthma. Data were collected from individuals referred to the tertiary care Eskişehir City Hospital Occupational Diseases Polyclinic over a 1‐year period, from January 1, 2024, to December 31, 2024. Ethical approval was obtained from the Eskişehir City Hospital Scientific Research Ethics Committee (decision number: ESH/BAEK 2025/89, dated January 07, 2025).

### 2.1 Study Design

This study employed a retrospective, descriptive design to provide an in‐depth examination of cases referred with suspected occupational asthma. The objective was to analyze demographic, clinical, diagnostic, and exposure‐related data to identify patterns and characteristics associated with occupational asthma within a defined cohort.

### 2.2 Study Population

**FIGURE 1:**
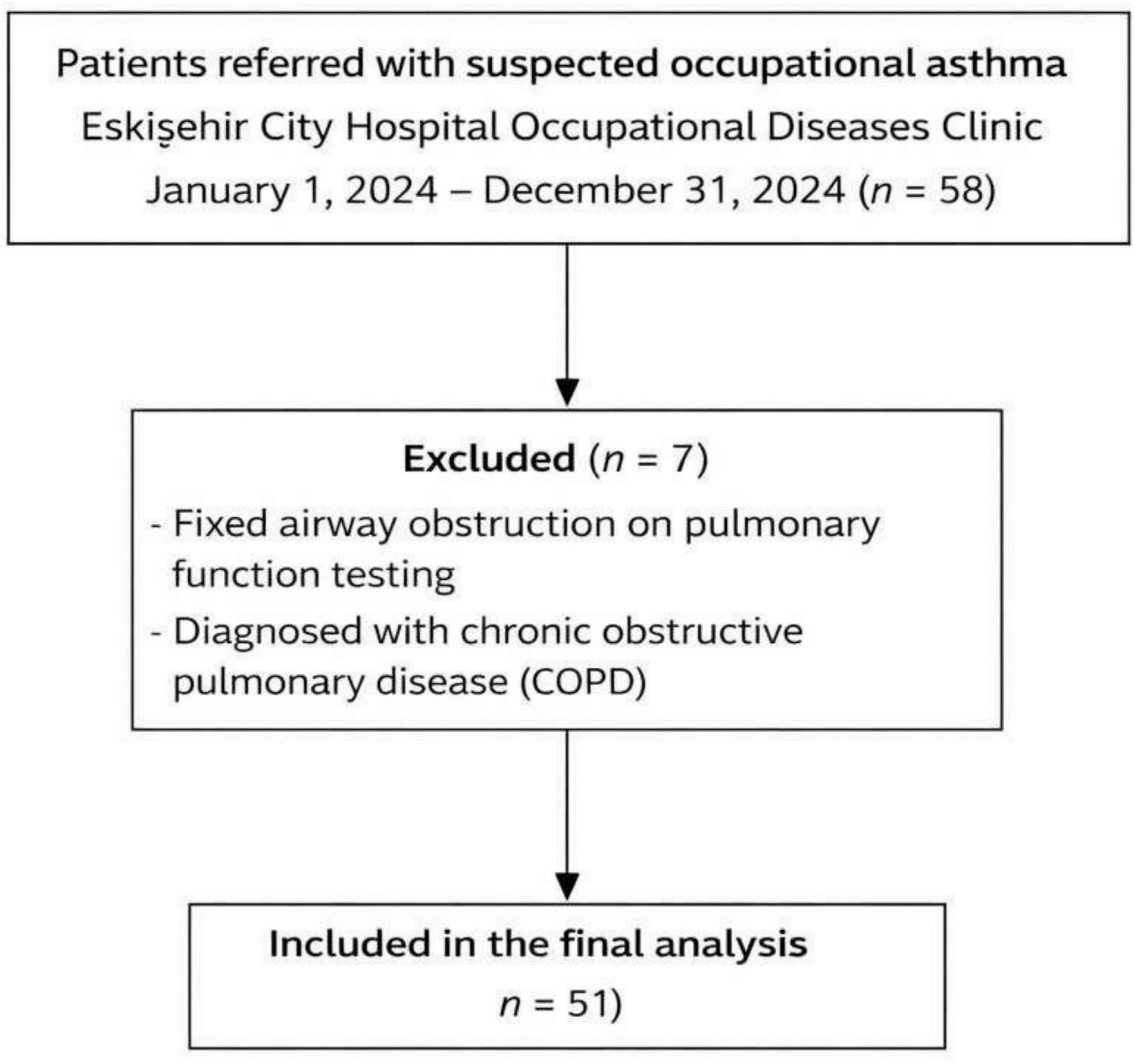
Flow diagram of patient selection and study population.

The study population consisted of individuals referred to the tertiary care Eskişehir City Hospital Occupational Diseases Polyclinic for suspected occupational asthma between January 1, 2024, and December 31, 2024. A total of 58 cases were referred with suspicion of occupational asthma. In seven cases, fixed airway obstruction findings were detected on pulmonary function testing, and these patients were diagnosed with chronic obstructive pulmonary disease and excluded from the study (Figure 1).

### 2.3 Inclusion Criteria

Individuals were included if they met the following criteria:

- The presence of asthma symptoms potentially associated with occupational exposures
- Asthma diagnosis supported by clinical evaluation, physical examination findings, objective pulmonary function tests, reversibility testing, and/or methacholine challenge testing
- Known or suspected exposure to occupational agents capable of triggering or exacerbating asthma
- Respiratory symptoms consistent with asthma and temporally related to the work environment

### 2.4 Exclusion Criteria

Exclusion criteria included the following:

- Individuals under 18 years of age
- Individuals with multiple occupational exposures (e.g., employment in more than one sector) to reduce confounding
- Cases with inadequate PEFR monitoring, defined as fewer than 2 weeks of monitoring or fewer than four measurements per day both at work and away from work

### 2.5 Data Collection

The study was conducted at Eskişehir City Hospital Occupational Diseases Clinic, a specialized center for diagnosing and reporting occupational diseases. Data were obtained from electronic medical records, e‐report systems, and archived outpatient records.

Demographic variables, including age, gender, smoking status, and occupational history, were recorded. Clinical data included symptom characteristics, duration, and the relationship between symptoms and workplace exposure. All patients completed a detailed asthma exposure questionnaire assessing both occupational and nonoccupational exposures.

### 2.6 Data Variables

The following variables were analyzed:

- Demographic characteristics: age, gender, and smoking status
- Clinical characteristics: respiratory symptoms, symptom duration, and comorbidities
- Exposure characteristics: type and duration of occupational exposure
- Diagnostic findings: spirometry, reversibility testing, methacholine challenge testing, and radiological findings
- PEFR monitoring results.

### 2.7 Laboratory and Respiratory Function Tests

#### 2.7.1 PEFR Monitoring

PEFR measurements were recorded at least four times daily over a minimum of 14 days during both work and away‐from‐work periods (Figure S1).

PEFR variability was calculated using the formula:

$$\left(\text{maximum PEFR}–\text{minimum PEFR}\right)/\text{maximum PEFR}\times 100$$

A variability exceeding 10% between work and nonwork periods was considered indicative of occupational asthma. Measurements obtained during workplace exposure and away from work were analyzed separately.

#### 2.7.2 Pulmonary Function Tests

Pulmonary function tests were performed using COSMED QUARK PFT (C03800‐01‐05) and related devices (Cosmed Pony FXR and Cosmed Q‐Box Body). Airway obstruction was defined as FEV_1_/FVC ≤ 70% according to ATS/ERS criteria [1, 16].

#### 2.7.3 Methacholine Challenge Test

Methacholine challenge testing was performed under standardized conditions in accordance with ATS/ERS recommendations [1, 16]. Methacholine was administered in doubling concentrations until a significant bronchial response was achieved or the maximum concentration was reached. A positive test was defined as a ≥ 20% decrease in FEV_1_ from baseline (PC20) [18].

To assess occupational relevance, testing was performed both during workplace exposure and after at least 15 days away from exposure. PC20 variability was defined as a clinically significant increase following exposure cessation [8, 18]. In this study, a threefold or greater increase in PC20 was used as a threshold.

In cases where methacholine challenge testing could not be performed due to low baseline lung function, diagnostic evaluation relied on PEFR variability combined with clinical and exposure history.

#### 2.7.4 IgE Assessment

Total IgE and specific IgE levels were measured to identify potential allergic sensitization to occupational allergens. Skin prick testing was performed when appropriate.

### 2.8 Diagnostic Definition

The diagnosis of occupational asthma was based on a combination of the following:

- Clinical history demonstrating a temporal relationship between symptoms and workplace exposure
- Objective evidence of variable airflow limitation (PEFR variability and/or methacholine PC20 variability)
- Improvement following removal from exposure

This approach is consistent with ATS/ERS recommendations and reflects real‐world clinical practice.

### 2.9 Statistical Analysis

Statistical analysis was performed using the Statistical Package for the Social Sciences (SPSS) for Windows version 20.0 (SPSS Inc., Chicago, IL, USA).

Categorical variables were expressed as frequencies and percentages, whereas continuous variables were presented as mean ± standard deviation.

Receiver operating characteristic (ROC) analysis was performed to evaluate the sensitivity, specificity, and optimal cutoff value of PC20 variability in predicting occupational asthma. The area under the curve (AUC) was calculated to assess diagnostic performance, and the Youden Index was used to determine the optimal threshold.

## 3 Results

### 3.1 Demographic Characteristics of the Study Population

A total of 51 cases suspected of occupational asthma were evaluated. The mean age of the participants was 38.14 ± 8.10 years, with 68.6% (*n* = 35) being male. Among the participants, 51% (*n* = 26) were current smokers, with an average smoking history of 8.41 ± 11.87 pack‐years. Household exposures included pet ownership in 13.7% (*n* = 7) of cases, mold and moisture exposure in 5.9% (*n* = 3), and workplace moisture exposure in 17.6% (*n* = 9). A history of allergies was reported in 7.8% (*n* = 4) of cases, whereas specific IgE positivity was detected in 38.7% (*n* = 12) of the 31 cases who underwent specific IgE testing. Two cases presented with occupational irritant contact dermatitis, confirmed by positive patch tests for epoxy, thiuram mix, titanium oxalate, and hydrate. Among the 13 cases who underwent prick testing, two showed sensitivity to tree pollen, house dust mites, and grass allergens (Table 1).

**TABLE 1:** Demographic and clinical characteristics of the study population.

Variable	Occupational asthma (*n* = 36)	Nonoccupational asthma (*n* = 15)	Total (*n* = 51)
Age (years), mean ± SD	37.03 ± 6.39	40.80 ± 11.03	38.14 ± 8.10
Gender, *n* (%)			
Female	7 (19.4)	9 (60.0)	16 (31.4)
Male	29 (80.6)	6 (40.0)	35 (68.6)
Duration of symptoms (months), mean ± SD	22.50 ± 32.98	12.67 ± 16.08	19.61 ± 29.23
Duration of exposure before symptom onset (years), mean ± SD	7.51 ± 5.94	6.92 ± 8.83	7.34 ± 6.82
Exposure agents, *n* (%)			
Low molecular weight (LMW) agents	30 (83.3)	11 (73.3)	41 (80.4)
Smoking (pack‐years), mean ± SD	7.31 ± 11.33	11.07 ± 13.09	8.41 ± 11.87
Smoking status, *n* (%)			
Nonsmoker	17 (47.2)	7 (46.7)	24 (47.1)
Current smoker	18 (50.0)	8 (53.3)	26 (51.0)
Ex‐smoker	1 (2.8)	0 (0.0)	1 (2.0)
Inhaled corticosteroid use, *n* (%)	7 (19.4)	4 (26.7)	11 (21.6)
Total IgE (IU/mL), mean ± SD	126.91 ± 224.76	169.11 ± 165.42	139.32 ± 208.33
Pulmonary function during exposure			
FEV_1_ (mL), mean ± SD	3.93 ± 1.04	3.53 ± 1.32	3.81 ± 1.13
FEV_1_ (%), mean ± SD	106.11 ± 24.97	107.87 ± 31.80	106.63 ± 26.84
FEV_1_/FVC, mean ± SD	76.94 ± 9.15	72.59 ± 10.29	75.66 ± 9.61
PC20 (mg/mL), mean ± SD	0.88 ± 1.45	4.25 ± 4.64	1.93 ± 3.20
Pulmonary function after exposure cessation			
FEV_1_ (mL), mean ± SD	4.68 ± 1.07	3.47 ± 1.17	4.38 ± 1.22
FEV_1_ (%), mean ± SD	120.68 ± 20.83	113.62 ± 29.46	119.07 ± 23.81
FEV_1_/FVC, mean ± SD	79.39 ± 6.70	72.08 ± 13.26	77.97 ± 8.85
PC20 (mg/mL), mean ± SD	11.71 ± 6.43	4.68 ± 6.34	10.16 ± 6.99
Environmental and clinical factors, *n* (%)			
Pet ownership at home	4 (11.1)	3 (20.0)	7 (13.7)
Mold/moisture at home	1 (2.8)	2 (13.3)	3 (5.9)
Mold/moisture at workplace	3 (8.3)	6 (40.0)	9 (17.6)
Pneumonia history	3 (8.3)	2 (13.3)	5 (9.8)
COVID‐19 history	7 (19.4)	4 (26.7)	11 (21.6)
Family history of asthma	4 (11.1)	2 (13.3)	6 (11.8)
High heavy metal exposure^a^	8 (22.2)	1 (6.7)	9 (17.6)
Bronchiectasis	0 (0.0)	1 (6.7)	1 (2.0)
Hypersensitivity pneumonitis	2 (5.6)	0 (0.0)	2 (3.9)
Pneumoconiosis	4 (11.1)	0 (0.0)	4 (7.8)
Allergic rhinitis	1 (2.8)	0 (0.0)	1 (2.0)
Previous asthma history	1 (2.8)	5 (33.3)	6 (11.8)
IgE‐dependent asthma	11 (30.6)	11 (73.3)	22 (43.1)

Abbreviations: FEV_1_, forced expiratory volume in 1 s; FVC, forced vital capacity; IgE, immunoglobulin E; LMW, low molecular weight; NOA, nonoccupational asthma; OA, occupational asthma; PC20, provocative concentration of methacholine causing a 20% fall in FEV_1_; SD, standard deviation.

^a^ Percentages are calculated among workers with documented exposure to metal fumes and dust.

The most commonly reported symptom was shortness of breath, with an average duration of 19.61 ± 29.23 months from symptom onset. The mean duration of occupational exposure before the onset of symptoms was 7.34 ± 6.82 years. Low molecular weight (LMW) agents were the most frequently reported occupational exposures, with nine cases reporting exposure to high molecular weight (HMW) agents (Tables 2 and 3).

**TABLE 2:** Distribution of Industries among the Study Population (*n* = 51).

Industry	Occupational Asthma (n = 36)	Nonoccupational asthma (*n* = 15)	Total (*n* = 51)
Furniture	2 (3.9%)	1 (2.0%)	3 (5.9%)
Textile	1 (2.0%)	0 (0.0%)	1 (2.0%)
Ceramic	7 (13.7%)	2 (3.9%)	9 (17.6%)
Foundry	5 (9.8%)	1 (2.0%)	6 (11.8%)
Plastic	1 (2.0%)	0 (0.0%)	1 (2.0%)
Metal	8 (15.7%)	1 (2.0%)	9 (17.6%)
Chicken and cattle breeding farm	2 (3.9%)	2 (3.9%)	4 (7.8%)
Mining	1 (2.0%)	0 (0.0%)	1 (2.0%)
Carton packaging industry	2 (3.9%)	0 (0.0%)	2 (3.9%)
Municipal park and garden cleaning services, landscape activities	1 (2.0%)	1 (2.0%)	2 (3.9%)
Manufacture of other builders' carpentry and joinery	2 (3.9%)	1 (2.0%)	3 (5.9%)
Manufacture of prepared feeds for farm animals	1 (2.0%)	0 (0.0%)	1 (2.0%)
Manufacture of chemicals and chemical products (laboratory)	2 (3.9%)	0 (0.0%)	2 (3.9%)
Municipal asphalt production plant	1 (2.0%)	0 (0.0%)	1 (2.0%)
Food industry	0 (0.0%)	2 (3.9%)	2 (3.9%)
Artificial marble cutting	0 (0.0%)	1 (2.0%)	1 (2.0%)
Manufacture of glass and glass products	0 (0.0%)	1 (2.0%)	1 (2.0%)
Cleaning activities	0 (0.0%)	2 (3.9%)	2 (3.9%)

**TABLE 3:** Distribution of occupational exposures (*n* = 51).

Classification of exposures	Occupational asthma (*n* = 36)	Nonoccupational asthma (*n* = 15)	Total (*n* = 51)
HMW agents			
Garbage dust	0 (0.0%)	1 (2.0%)	1 (2.0%)
Paper and cellulose dust	2 (3.9%)	0 (0.0%)	2 (3.9%)
Animal epithelia	1 (2.0%)	2 (3.9%)	3 (5.9%)
Meadow, grass, herb	1 (2.0%)	0 (0.0%)	1 (2.0%)
Animal feed dust	1 (2.0%)	0 (0.0%)	1 (2.0%)
Wool yarn dust	1 (2.0%)	0 (0.0%)	1 (2.0%)
LMW agents			
Wood dust	4 (7.8%)	1 (2.0%)	5 (9.8%)
Dyes and solvents	1 (2.0%)	1 (2.0%)	2 (3.9%)
Ammonia, nitrogen, and hydrazine gases	2 (3.9%)	1 (2.0%)	3 (5.9%)
Detergents	0 (0.0%)	3 (5.9%)	3 (5.9%)
Carbon, perlite, silica, and silicate dust	10 (19.6%)	1 (2.0%)	11 (21.6%)
Welding fumes	3 (5.9%)	1 (2.0%)	4 (7.8%)
Chemicals and chemical compounds	1 (2.0%)	2 (3.9%)	3 (5.9%)
Chlorine	1 (2.0%)	0 (0.0%)	1 (2.0%)
Metal fume and dust	4 (7.8%)	1 (2.0%)	5 (9.8%)
Metal dust with chemicals and oils	2 (3.9%)	0 (0.0%)	2 (3.9%)
Methyl sulfite, sulfur, and soda mix	1 (2.0%)	0 (0.0%)	1 (2.0%)
Bitumen	1 (2.0%)	0 (0.0%)	1 (2.0%)
Polyurethane and epoxy	0 (0.0%)	1 (2.0%)	1 (2.0%)

Abbreviations: HMW, high molecular weight; LMW, low molecular weight; NOA, nonoccupational asthma; OA, occupational asthma.

### 3.2 Demographic Characteristics of Occupational Asthma Cases

Among the 51 cases, 36 (70.6%) were diagnosed with occupational asthma (OA). The mean symptom duration in OA cases was 22.50 ± 32.98 months, and the mean duration of exposure to the suspected causative agent was 7.51 ± 5.94 years. Symptom duration and exposure time were both longer in the OA group compared to nonoccupational asthma (NOA) cases. Total IgE levels were higher in NOA cases than in OA cases (OA: 126.91 ± 224.76 IU/mL, NOA: 169.11 ± 165.42 IU/mL). Specific IgE positivity was observed in 19.4% (*n* = 7) of OA cases. IgE‐dependent occupational asthma was identified in 30.6% (*n* = 11) of OA cases (Table 1). Two cases had positive patch tests for epoxy, thiuram mix, titanium oxalate, and hydrate. Among patients exposed to LMW agents, which included hydrazine, silica, silicates, chlorine, metals, and isocyanates, 16.7% (*n* = 6) exhibited IgE‐mediated asthma. Nonimmunologic mechanisms contributed to 83.3% (*n* = 30) of OA cases.

During occupational exposure, PC20 levels were lower in OA cases but improved after exposure cessation. In 13.9% (*n* = 5) of OA cases, methacholine challenge testing could not be performed due to FEV_1_ levels below 70%, and diagnosis was based on positive PEFR variability. Three cases in which PEFR variability could not be assessed due to workplace changes were diagnosed based on PC20 variability. The mean PC20 value increased from 0.88 ± 1.45 mg/mL during exposure to 11.71 ± 6.43 mg/mL after exposure cessation, indicating a marked reduction in bronchial hyperresponsiveness following removal from exposure. In the OA group, both PEFR and PC20 variability were positive in 25% (*n* = 9) of 36 cases (Table 4).

**TABLE 4:** Distribution of PEFR and PC20 variability.

Category	Occupational asthma (*n* = 36)	Nonoccupational asthma (*n* = 15)	Total (*n* = 51)
PEFR (−) and PC20 (−)	0 (0.0%)	9 (60.0%)	9 (17.6%)
PEFR (−) and PC20 (+)	16 (44.4%)	0 (0.0%)	16 (31.4%)
PEFR (+) and PC20 (+)	9 (25.0%)	1 (6.7%)	10 (19.6%)
PEFR not performed^a^ and PC20 (+)	3 (8.3%)	0 (0.0%)	3 (5.9%)
PEFR (+) and PC20 (−)	3 (8.3%)	1 (6.7%)	4 (7.8%)
PEFR (+) and NSBPT not performed^b^	5 (13.9%)	1 (6.7%)	6 (11.8%)
PC20 (+), PEFR not available^c^	0 (0.0%)	2 (13.3%)	2 (3.9%)
Testing discontinued^c^	0 (0.0%)	1 (6.7%)	1 (2.0%)

Abbreviations: NOA, nonoccupational asthma; NSBPT, nonspecific bronchial provocation test; OA, occupational asthma; PC20, provocative concentration of methacholine causing a 20% fall in FEV_1_; PEFR, peak expiratory flow rate.

^a^ PEFR monitoring could not be performed due to resignation or job change.

^**b**^ Methacholine challenge test (NSBPT) could not be performed due to baseline FEV_1_ < 70%.

^**c**^ Cases were classified as nonoccupational asthma due to incomplete evaluation of occupational causality.

**FIGURE 2:**
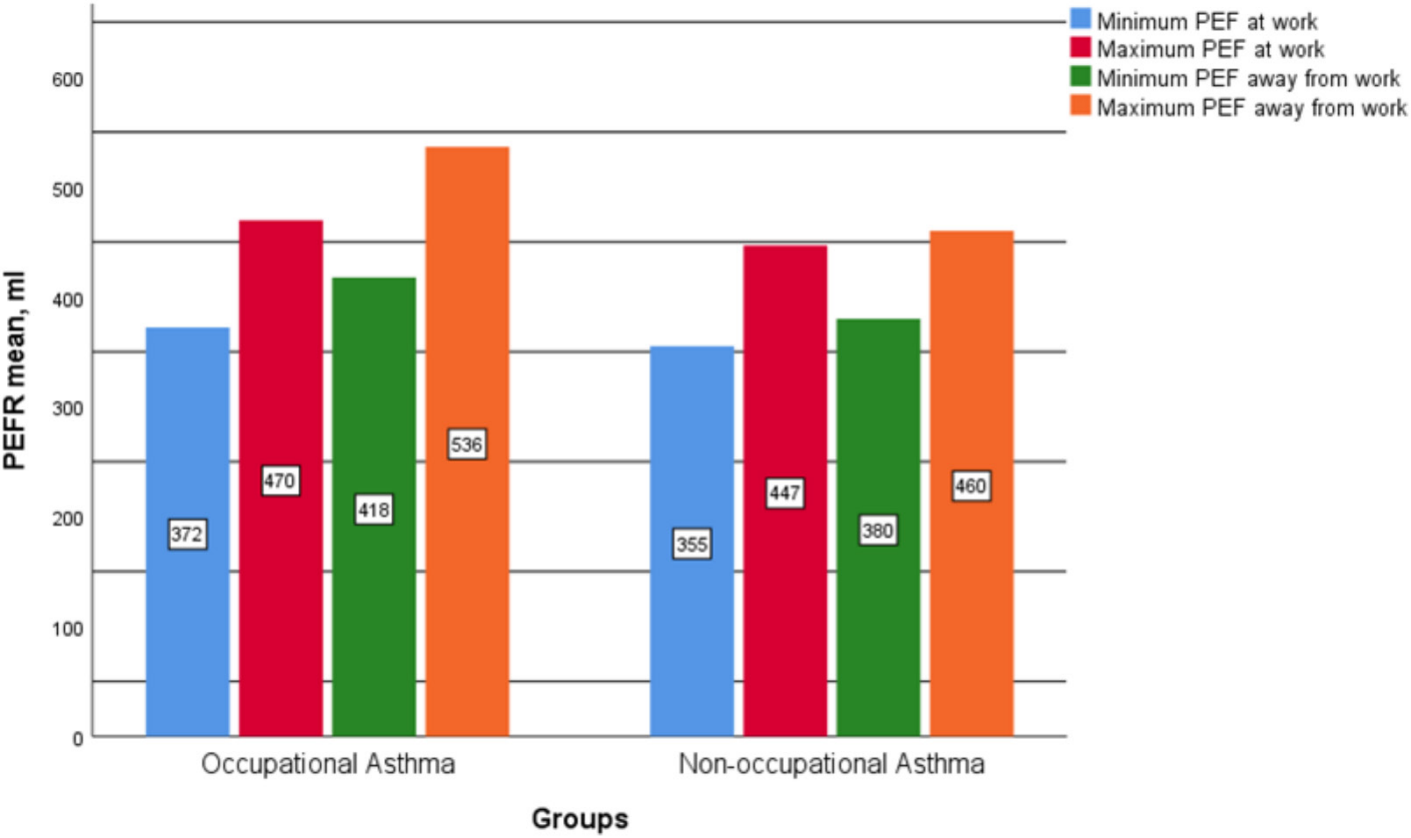
Comparison of mean PEFR values between OA and NOA cases.

FEV1/FVC ratios were lower in OA cases during exposure but improved after exposure cessation, whereas NOA cases showed no significant change. In 20 cases, NSBPT results turned negative following exposure cessation (Table 1). Figure 2 illustrates changes in PEFR values in OA and NOA patients.

The majority of occupational asthma cases were linked to the metal and foundry industry, followed by the ceramics sector. The most common exposure was silica and silicates (27.8%, *n* = 10). Welder's asthma was identified in 8.3% (*n* = 3) of cases. Additionally, four individuals had wood dust exposure due to work in wooden mold production and pallet manufacturing, though only two were employed in the furniture industry. A detailed breakdown of industries and exposures is presented in Tables 2 and 3.

## 4 Discussion

The diagnosis of OA remains complex due to several challenges, including the variability in individual risk factors, the multifactorial nature of its pathogenesis, and the frequent inability to identify specific workplace triggers. Moreover, the absence of established exposure threshold values complicates accurate diagnosis [16]. A crucial element in diagnosing OA is demonstrating bronchial hyperresponsiveness in the workplace. Although SBPT is considered the gold standard for diagnosis, a negative result does not definitively exclude OA [19]. Furthermore, multiple diagnostic tools are available, but the utility of each varies, especially in terms of sensitivity and specificity. In this study, we assessed the role of PEFR monitoring and methacholine PC20 variability in diagnosing OA and evaluated their respective strengths and limitations.

### 4.1 PEFR Monitoring and its Role in Diagnosis

PEFR monitoring remains a widely used diagnostic tool in many countries, including Turkey. However, the sensitivity and specificity of PEFR variability have yielded inconsistent results across studies [20, 21]. Côté et al. [22] demonstrated that combining PEFR with clinical history had a sensitivity of 100% but a specificity of only 45%. When combined with methacholine PC20 variability, the specificity increased to 67%. Moore et al. [20] reported that serial PEFR measurements had a sensitivity of 82% and a specificity of 88% in diagnosing OA. These findings indicate that the sensitivity and specificity of PEFR monitoring can vary, and when used alone, it may lead to missed cases. Our study also revealed that relying solely on PEFR could lead to an underestimation of OA diagnosis. Only 8.3% of occupational asthma cases were identified based solely on PEFR variability, whereas a higher proportion showed PEFR positivity when combined with other diagnostic findings. These findings highlight the limited diagnostic value of PEFR when used alone and support the use of combined diagnostic approaches in occupational asthma.

Despite the limitations of PEFR, the method remains useful when combined with other diagnostic approaches, such as clinical history and laboratory findings. In certain cases where more comprehensive tests such as SBPT are contraindicated or unavailable, PEFR variability may still offer valuable diagnostic insights. Furthermore, PEFR variability is particularly helpful in scenarios where the patient's exposure to workplace irritants has ceased after an asthma diagnosis, and monitoring through NSBPT is no longer possible.

The relatively low sensitivity of PEFR observed in our study (39.6%) compared to previous reports may be explained by differences in patient populations, variability in workplace exposure conditions, reliance on self‐reported measurements, and the use of nonelectronic PEFR devices, which may introduce measurement bias.

### 4.2 PC20 Variability in Diagnosis

**FIGURE 3:**
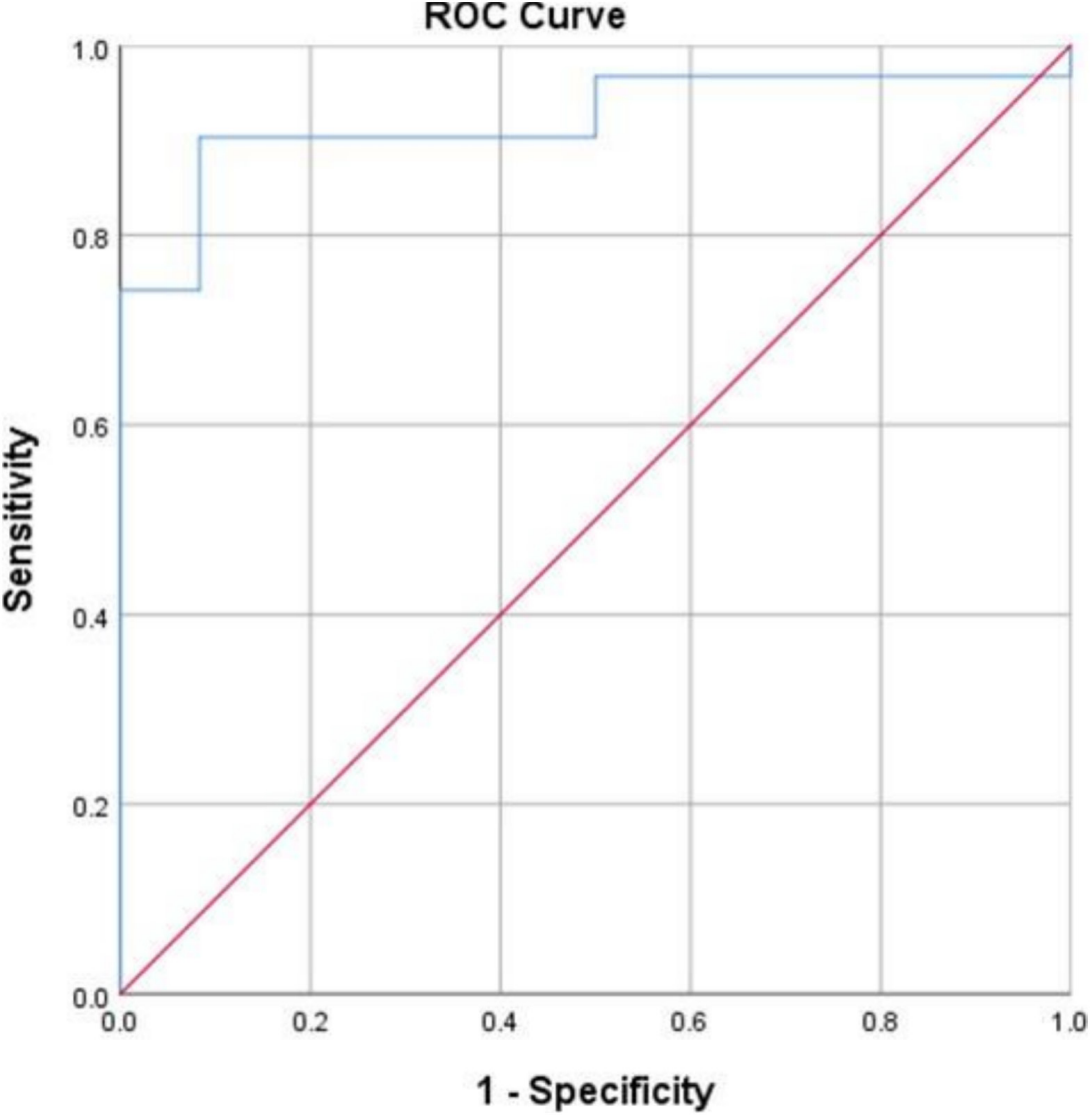
ROC curve of PC20 variability for diagnosing occupational asthma.

In contrast to PEFR, PC20 variability demonstrated high sensitivity and specificity in our study. PC20 variability was positive in 77.8% of cases and had a sensitivity of 90% and a specificity of 92% (Table 5, Figure 3). These results are consistent with findings from previous studies, which have also highlighted the utility of methacholine challenge tests in diagnosing OA [23]. Notably, the high sensitivity and specificity of PC20 variability in our study suggest that it could serve as a reliable alternative or complementary diagnostic tool when PEFR monitoring is impractical or unreliable, particularly in the context of workplace exposures that cease following the diagnosis of asthma.

**TABLE 5:** Sensitivity and specificity of PC20 variability for diagnosing occupational asthma.

Parameter	AUC (95% CI)	Cutoff	Sensitivity	Specificity	*p*
PC20 variability	0.922 (0.839–1.000)	2.81	0.90	0.92	0.001

Abbreviations: AUC, area under the curve; CI, confidence interval; PC20, provocative concentration of methacholine causing a 20% fall in FEV_1_.

Additionally, the ability to demonstrate NSBPT negativity after cessation of exposure is critical for confirming the link between work‐related exposures and asthma. This aspect of our study aligns with previous research, which suggests that the absence of bronchial hyperresponsiveness after exposure cessation is a valuable diagnostic criterion for OA [19].

### 4.3 Limitations of PEFR and Variability in its Sensitivity and Specificity

Although PEFR monitoring is commonly employed in many countries, including Turkey, it is susceptible to several limitations. In many workplaces, PEFR measurements are not consistently monitored by occupational physicians, and individuals often record their own PEFR values, leading to variability in the data. Nonelectronic PEFR devices, which are frequently used in clinical settings, do not provide digital records, further exacerbating this issue [23]. PEFR is significantly influenced by respiratory effort [24]. Even with training, the recorded PEFR values tend to differ from those measured in hospital settings, leading to inconsistencies in the interpretation of results. In our study, some OA cases were diagnosed solely based on PEFR variability, corroborated by exposure history and clinical findings. This demonstrates the utility of PEFR, despite its limitations, in specific circumstances where NSBPT is contraindicated or unavailable.

### 4.4 Sector‐Specific Exposures and Role in Occupational Asthma

According to Côté et al. [22], workplace mold exposure significantly increases the frequency of asthma exacerbations. Our study is one of the few that also evaluated nonoccupational workplace factors such as humidity, dampness, and mold that could cause asthma [19, 25]. In a study involving 999 employees, it was determined that exposure to mold in the workplace increased asthma attacks by seven times [26]. Although specific IgE tests for mold, Aspergillus, and inhalants were negative, three cases reported humidity and dampness in their work environment. Three cases diagnosed with OA kept poultry at home, and one kept a cat. Our study is novel in that it thoroughly examines both occupational and environmental exposures that could cause asthma, despite sector differences.

Our study also highlights the significant role of various occupational exposures in the development of OA. The heterogeneity of exposures in our study population, primarily from the ceramic, metal, and foundry sectors, provided valuable insights into the diverse triggers of OA. These sectors are known to expose workers to LMW agents, which are often responsible for triggering asthma [27, 28]. In our study, a high prevalence of asthma was observed in these sectors, particularly in workers exposed to LMW agents. However, our study is one of the few to also consider nonoccupational factors, such as humidity, mold, and dampness, which could contribute to asthma development.

Furthermore, our study identified cases where asthma coexisted with contact dermatitis, particularly in individuals exposed to irritants like plaster dust, epoxy, and chemicals used in metal surface cleaning. These cases reinforce the need for clinicians to consider the possibility of OA accompanying other occupational diseases, such as contact dermatitis [16].

### 4.5 Irritant‐Induced Asthma and Heavy Metal Exposure

In addition to asthma induced by immunologic agents, our study found several cases of irritant‐induced asthma, particularly in workers exposed to chlorine, hydrazine, and welding fumes. Although irritant‐induced asthma is less frequently studied than allergic asthma [20, 29], our findings contribute to the understanding of irritant‐induced OA, which is prevalent in industries such as metal and ceramics. Notably, some of our cases were exposed to elevated levels of heavy metals, which have been linked to respiratory dysfunction in previous studies [30]. These findings highlight the importance of considering both immunologic and irritant‐induced mechanisms in the pathogenesis of OA.

### 4.6 Differentiating Between OA and Other Respiratory Diseases

**FIGURE 4:**
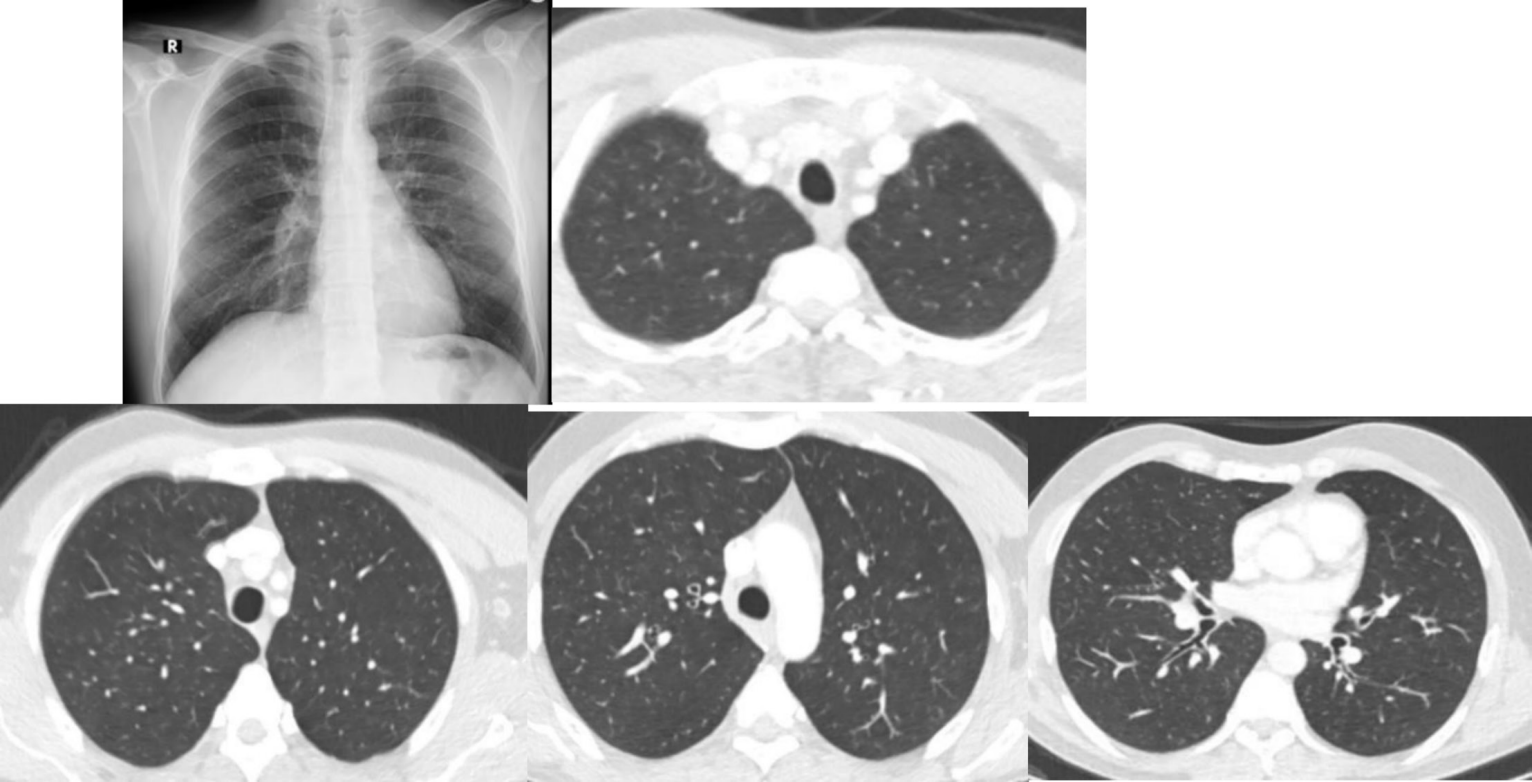
Imaging findings suggestive of extrinsic allergic alveolitis in a patient with occupational asthma.

The complexity of diagnosing OA is further illustrated by the challenge of differentiating OA from other respiratory diseases, such as extrinsic allergic alveolitis. In our study, we identified cases with radiological findings suggestive of extrinsic allergic alveolitis, which were associated with workplace exposures to wool yarn production and wooden mold manufacturing (Figure 4). Differentiating between these two conditions is often difficult, and our study contributes to the body of knowledge on the overlap between OA and other respiratory diseases [19, 31].

### 4.7 Biases and Limitations

This study presents several potential biases and limitations that should be considered when interpreting the findings. The study primarily focuses on individuals from specific sectors, namely ceramics, metalworking, and foundries, which are industries with high exposure to LMW agents known to trigger asthma. Although this provides valuable insights into the relationship between these industries and occupational asthma, it could lead to selection bias, as these sectors may not be representative of all industries. The findings may thus be particularly relevant to these industries, limiting the generalizability of the results to other sectors with different exposure profiles.

The study includes a heterogeneous group of participants, exposed to various occupational and environmental factors. Although this diversity provides a broad perspective on asthma triggers, it also complicates the diagnosis and interpretation of results. The varying exposure types (both occupational and environmental) and asthma mechanisms (immunologic vs. irritant‐induced) may influence the diagnostic accuracy of tests such as PEFR and PC20 variability, making it difficult to draw uniform conclusions across such a diverse group.

PEFR monitoring is a common diagnostic tool, but it is subject to several limitations, particularly when measurements are self‐reported by the workers. In settings where PEFR values are recorded by the individuals themselves using nonelectronic devices, the accuracy of these measurements can be compromised. Even with proper training, PEFR values recorded in nonclinical settings often differ from those measured in a hospital environment. This inconsistency can lead to potential misclassification of asthma cases and complicates the diagnostic process, especially when PEFR is used as the sole criterion.

One of the limitations of this study is the absence of SBPT as a reference standard for diagnosing OA. Although SBPT is considered the gold standard for diagnosing OA, its absence in this study means that the sensitivity and specificity of the other diagnostic tools (PEFR and PC20 variability) cannot be fully validated against the gold standard.

The study also considers environmental factors, such as humidity, dampness, and mold, which may contribute to asthma development. However, assessing the impact of environmental exposures is inherently challenging due to the difficulty in accurately measuring home or nonoccupational exposures over a prolonged period. Despite including these factors, the study may not have fully accounted for the extent to which they contributed to asthma development, as the focus remained on occupational exposures.

The study includes a relatively small sample size, particularly in relation to specific subgroups such as those exposed to heavy metals or particular sectors (ceramics, metal, foundry, etc.). A smaller sample size limits the statistical power of the study and may reduce the reliability and generalizability of the findings. To confirm the study's conclusions, further research with a larger sample size is needed to enhance the statistical validity and robustness of the results. In conclusion, several biases and limitations should be acknowledged in this study.

## 5 Conclusion

This study underscores the importance of a multifactorial approach to diagnosing occupational asthma, incorporating occupational exposure history, clinical evaluation, and diagnostic tests such as PEFR and PC20 variability. The findings highlight both the limitations and complementary value of PEFR monitoring, particularly when used alongside other diagnostic tools, and emphasize the reliability of PC20 variability in diagnosing occupational asthma. Furthermore, the study demonstrates the significant role of both occupational and environmental exposures in the development of asthma, especially in high‐risk industries involving respiratory sensitizers.

Our study is novel in that it comprehensively evaluates both occupational and environmental exposures within a real‐world clinical setting. Future studies with larger populations are warranted to further validate these findings. A multidisciplinary approach involving occupational health specialists, employers, and policymakers is essential to prevent occupational asthma and reduce respiratory risks in the workplace.

## Author Contributions

Conceptualization: B.A. Methodology: B.A. Software: B.A. and G.Y. Validation: B.A. and G.Y. Formal analysis: B.A. Investigation: B.A. Resources: G.Y. Data curation: B.A. and G.Y. Writing – original draft preparation: B.A. Writing – review and editing: B.A. and G.Y. Visualization: B.A. Supervision: B.A. Project administration: B.A. All authors have read and agreed to the published version of the manuscript.

## Funding

The authors have nothing to report.

## Ethics Statement

The study was conducted in accordance with the Declaration of Helsinki and approved by the Institutional Ethics Committee of the Eskişehir City Hospital Scientific Research Ethics Committee protocol code ESH/BAEK 2025/89 and dated of approval January 07, 2025 for studies involving humans. Patient consent was waived due to the retrospective analysis of existing anonymized data that does not involve any direct interventions or interactions with human participants. All authors approved the manuscript to publish.

## Conflicts of Interest

The authors declare no conflicts of interest.

## Supporting information


**Figure S1:** The PEF monitoring form used in the Eskişehir City Hospital Occupational Diseases Clinic requires that values at work be noted with a blue pen and at the place of duty, values away from work with a red pen and no indoor exposures be excluded.

## Data Availability

The data that support the findings of this study are available from the corresponding author upon reasonable request.
