# Supplementary figures and images for: Using Multiple Diagnostic Methods for Occupational Asthma Assessment
Source: Clin Respir J. 2026 May 13;20(5):e70197. doi: 10.1111/crj.70197 (PMC13170094; doi:10.1111/crj.70197)

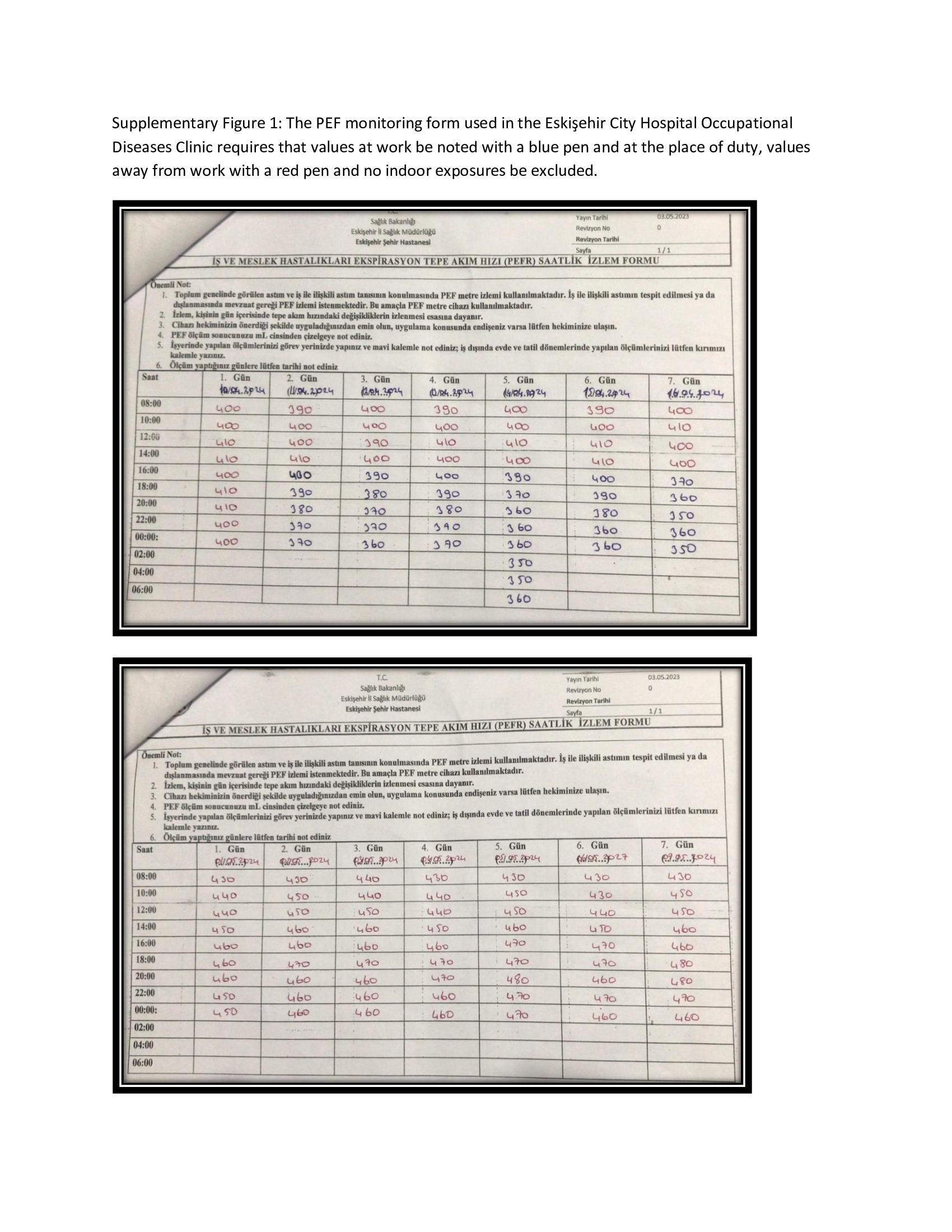

Supplement: Supplementary file 1 — Figure S1: The PEF monitoring form used in the Eskişehir City Hospital Occupational Diseases Clinic requires that values at work be noted with a blue pen and at the place of duty, values away from work with a red pen and no indoor exposures be excluded. [file CRJ-20-e70197-s001.jpg]
